# Supplementary material for: Multilocus phylogeny and historical biogeography of Hypostomus shed light on the processes of fish diversification in La Plata Basin
Source: Sci Rep. 2021 Mar 3;11:5073. doi: 10.1038/s41598-021-83464-x (PMC7930046; doi:10.1038/s41598-021-83464-x)
Supplement: Supplementary file 1 — Supplementary Information [file 41598_2021_83464_MOESM1_ESM.docx]

**Supplementary information**

**Multilocus phylogeny and historical biogeography of *Hypostomus* shed light on the processes of fish diversification in La Plata Basin**

**SHORT RUNNING TITLE**: *Hypostomus* diversification in La Plata Basin

Yamila P. Cardoso^1^*^†^| Luiz Jardim de Queiroz^2†^| Ilham A. Bahechar^2^| Paula E. Posadas^1^| Juan I. Montoya-Burgos^2,3^

^1^ Laboratorio de Sistemática y Biología Evolutiva, Facultad de Ciencias Naturales y Museo, Universidad Nacional de La Plata, Paseo del Bosque S/N, B1900FWA, La Plata, Buenos Aires, Consejo Nacional de Investigaciones Científicas y Técnicas, Argentina.

^2^ Department of Genetics and Evolution, University of Geneva, 30 quai Ernest Ansermet, 1211, Geneva 4, Switzerland.

^3^ Institute of Genetics and Genomics in Geneva (IGE3), University of Geneva, Geneva, Switzerland.

^†^ These authors contributed equally to this work

*Author to whom correspondence should be addressed: Tel: +54 221 422-8451 int. 140, e-mail: [yamilapcardoso@gmail.com](mailto:yamilapcardoso@gmail.com), ORCID: [https://orcid.org/0000-0003-3497-4359](https://mc.manuscriptcentral.com/jbi%2523)

Table S1. Full list of analysed specimens, collection code, GenBank accession numbers, collection locality, and habitat preference code (see main text). All the GenBank accession numbers starting with MK correspond to sequences newly generated for this study.

| Species | Collection  code | Genbank number (Dloop/COI/Rag1/HAMzbtb10-3/Hodz3) | Location  (Country, river system) | Habitat preferences |
| --- | --- | --- | --- | --- |
| Hypostomus affinis | BR1254 | AJ318358/MK959853/MK959930/MK959980/MK960036 | Brazil, Agua Santa River | 1234 |
| Hypostomus albopunctatus | MCP37990 | AJ318379/MK959865/MK959942/MK959992/MK960048 | Brazil, Iguacu River | 23 |
| Hypostomus ancistroides | BR98696 | AJ318369/MK959857/MK959934/MK959984/MK960040 | Brazil, Rio Grande River Basin | 3 |
| Hypostomus arecuta | AG198 | JF290445/MK959840/MK959916/MK959967/MK960024 | Argentina, Middle Parana River | 3 |
| Hypostomus asperatus | BR974 | AJ318370/MK959854/MK959931/MK959981/MK960037 | Brazil, Bateia River | 3 |
| Hypostomus aspilogaster | DF023 | AJ318375/MK959860/MK959937/MK959987/MK960043 | Brazil, Rio Grande do Sul | 34 |
| Hypostomus borelli | BO14260 | MK959903/MK959848/MK959925/MK959975/- | Bolivia, Pilcomayo River | 1 |
| Hypostomus boulengeri | AR11609 | JX290097/MK959846/MK959923/MK959973/MK960030 | Argentina, Paraguay River | 34 |
| Hypostomus cochliodon | 1154 | JF290476/MK959839/MK959915/MK959966/MK960022 | Argentina, Middle Parana River | 2 |
| Hypostomus commersoni | YC09118 | MK959895/MK959883/MK959959/MK960012/MK960067 | Argentina, Rio de la Plata | 1234 |
| Hypostomus cordovae | AR111215 | KX852408/MK959844/MK959921/MK959971/MK960028 | Argentina, Segundo River | 12 |
| Hypostomus derby | YC10316 | JF290447/MK959885/MK959961/MK960014/MK960069 | Argentina, Middle Iguazu River | 1 |
| Hypostomus ericae | BR1013 | AJ318347/MK959849/MK959926/MK959976/MK960032 | Brazil, Maranhao River Basin | 2 |
| Hypostomus fonchii | PE08034 | MK959900/MK959868/MK959945/MK959995/MK960051 | Peru, Huecamayo River | 12 |
| Hypostomus formosae | AR11207 | JX290093/MK959845/MK959922/MK959972/MK960029 | Argentina, Paraguay River | 2 |
| Hypostomus hemirus | GY04333 | MK959898/MK959864/MK959941/MK959991/MK960047 | Guyane, Siparuni donwstream | 1 |
| Hypostomus hondae | VZ94 | AJ318348/MK959888/MK959956/MK960009/MK960064 | Venezuela, Muyapa River | 1 |
| Hypostomus interruptus | BR1161 | AJ412846/MK959851/MK959928/MK959978/MK960034 | Brazil, Ribeira do Iguape River | 23 |
| Hypostomus isbrueckeri | MCP41476 | AJ318376/MK959866/MK959943/MK959993/MK960049 | Brazil, Jacui River | 3 |
| Hypostomus laplatae | YC09031 | KX852412/MK959881/MK959957/MK960010/MK960065 | Argentina, Rio de la Plata | 4 |
| Hypostomus latifrons | PY8008 | AJ318378/MK959873/MK959950/MK960000/MK960056 | Paraguay, Salado River | 34 |
| Hypostomus luteomaculatus | AG200 | JF290467/MK959841/MK959917/MK959968/MK960025 | Argentina, Middle Parana River | 34 |
| Hypostomus luteus | UR007 | AJ318374/MK959876/MK959953/MK960003/MK960059 | Brazil, Rio Uruguay River | 2 |
| Hypostomus microstomus | 1153 | JF290461/MK959838/MK959914/MK959965/MK960021 | Argentina, Middle Parana River | 2 |
| Hypostomus mutucae | Aqua9 | MK959894/MK959891/MK959920/MK960019/MK960072 | Brazil, Upper Paraguay River | 12 |
| Hypostomus myersi | YC10256 | AJ318355/MK959884/MK959960/MK960013/MK960068 | Argentina, Middle Iguazu River | 1 |
| Hypostomus nigromaculatus | Tib12 | AJ318355/MK959875/MK959952/MK960002/MK960058 | Brazil, Tibaji River | 2 |
| Hypostomus oculeus | PE08441 | MK959897/MK959871/MK959948/MK959998/MK960054 | Peru, Loreto | 1 |
| Hypostomus plecostomoides | VZ58 | AJ318349/MK959889/MK959955/MK960008/MK960063 | Venezuela, Apure/Masparo Bassin | 2 |
| Hypostomus plecostomus | SUJM047 | MK959905/MK959874/MK959951/MK960001/MK960057 | Suriname | 34 |
| Hypostomus regani | AG354 | MK959907/MK959843/MK959919/MK959970/MK960027 | Argentina, Middle Parana River | 1234 |
| Hypostomus sp. 207 | AG207 | MK959908/MK959842/MK959918/MK959969/MK960026 | Argentina, Middle Parana River | 12 |
| Hypostomus aff. plecostomus . | BO14052 | MK959904/MK959861/MK959938/MK959988/MK960044 | Bolivia, Rio Grande | 12 |
| Hypostomus sp 1100. | BR1100 | -/MK959892/MK960017/-/MK960073 | Brazil, Tocantins River | 2 |
| Hypostomus sp. 1211 | BR1211 | MK959906/MK959847/MK959924/MK959974/MK960031 | Brazil, Tiete River | 2 |
| Hypostomus sp. 219 | BR98219 | AJ412834/MK959850/MK959927/MK959977/MK960033 | Brazil, Itapicuru River | 3 |
| Hypostomus sp. 678 | BR98678 | AJ315764/MK959867/MK959944/MK959994/MK960050 | Brazil, Tiete River | 2 |
| Hypostomus sp. 699 | BR98699 | MK959896/MK959869/MK959946/MK959996/MK960052 | Brazil, Rio Grande | 2 |
| Hypostomus sp. 751 | BR98751 | MK959901/MK959870/MK959947/MK959997/MK960053 | Brazil, Sao Francisco River | 3 |
| Hypostomus sp. 2150 | FHN2150 | MK959899/MK959872/MK959949/MK959999/MK960055 | Argentina, Bermejo River | 12 |
| Hypostomus sp. 44495 | MCP44495 | AJ412848/MK959855/MK959932/MK959982/MK960038 | Brazil, Upper Paraguay River | 2 |
| Hypostomus sp. 47069 | MCP47069 | AJ412839/MK959859/MK959936/MK959986/MK960042 | Brazil, Upper Parana River | 2 |
| Hypostomus sp. 47195 | MCP47195 | AJ412844/MK959852/MK959929/MK959979/MK960035 | Brazil, Upper Parana River | 2 |
| Hypostomus sp. PdR36 | PdR36 | AJ412836/MK959856/MK959933/MK959983/MK960039 | Peru, Ucayali River | 2 |
| Hypostomus sp. 221 | PE08221 | AJ412835/MK959858/MK959935/MK959985/MK960041 | Peru, Monzon River | 2 |
| Hypostomus sp. 269 | PE08269 | -/MK959890/-/MK960018/- | Peru | 2 |
| Hypostomus sp. 700 | PE08700 | -/MK959893/-/MK960020/- | Peru, Neshua River near Neshua | 2 |
| Hypostomus spiniger | YC09091 | MG457224/MK959882/MK959958/MK960011/MK960066 | Argentina, Uruguay River | 34 |
| Hypostomus taphorni | GY04173 | MK959902/MK959863/MK959940/MK959990/MK960046 | Guyane, Sawarab bridge | 2 |
| Hypostomus ternetzi | YC164 | JF290462/MK959887/MK959963/MK960016/MK960071 | Argentina, Middle Parana River | 23 |
| Hypostomus uruguayensis | YC10356 | MK959909/MK959886/MK959962/MK960015/MK960070 | Argentina, Middle Parana River | 1234 |
| Hypostomus watwata | GF99162 | AJ318352/MK959862/MK959939/MK959989/MK960045 | French Guiana, Oyapok River | 5 |
| Outgroups | | | | |
| Aphanatorulus ammophilus | VZ142 | AJ318346/MK959879/MK959913/MK960006/MK959879 | Venezuela, San Carlos River, near Las Vegas | 2 |
| Hemiancistrus fuliginosus | UR008 | AJ318359/MK959877/MK959954/MK960004/MK960060 | Brazil, Uruguay River | 3 |
| Pterygoplichthys multiradiatus | VZ119 | AJ318361/MK959878/MK959912/MK960005/MK960061 | Venezuela, Aguaro River | 3 |
| Pterygoplichthys scrophus | 966 | AJ318362/MK959837/MK959910/MK959964/MK960023 | Peru, Loreto, Ucayali, Marañon | 2 |
| Pterygoplichthys zuliaensis | VZ4 | AJ318360/MK959880/MK959911/MK960007/MK960062 | Venezuela, Escalante River | 3 |

**Table S2**. Primers used to amplify the five markers, the sequence length of the amplified DNA fragment (bp) and the number of phylogenetically informative bp (Infor bp) .

| **Mitochondrial marker** | **Primers name and sequences (5'-3')** | **References** | **bp/**  **Infor bp** |
| --- | --- | --- | --- |
| Control region | DLAIII: TATTTAAAGRCATAATCTCTTGAC | [^14^](http://f1000.com/work/citation?ids=7175088&pre=&suf=&sa=0) | 592/197 |
|  | HgDL-R: WTGCKARTATGTGCCGYYTG | [^14^](http://f1000.com/work/citation?ids=7175088&pre=&suf=&sa=0) |  |
| Cytochrome oxidase I | SilCOI-D: GGTCAACAAATCATAAAGATATTGG | [^43^](http://sciwheel.com/work/citation?ids=8524955&pre=&suf=&sa=0) | 825/157 |
|  | SilCOI-R: TAAACTTCAGGGTGACCAAA AATCA | [^43^](http://sciwheel.com/work/citation?ids=8524955&pre=&suf=&sa=0) |  |
| **Nuclear marker** |  |  |  |
| Rag 1 | F74: TTTCGGAATGGAAGTTTAAGCTsTTTCG | [^87^](http://f1000.com/work/citation?ids=7175434&pre=&suf=&sa=0) | 1345/62 |
|  | R1333: GTCAAACACACAGACTTCACATC | [^87^](http://f1000.com/work/citation?ids=7175434&pre=&suf=&sa=0) |  |
|  | F74-iD: TGATTCTTCAGCTCTCTCAMC | [^43^](http://sciwheel.com/work/citation?ids=8524955&pre=&suf=&sa=0) |  |
|  | R1333-ir: CAAGTGAGAGAGGGTAAGCAG | This study |  |
| HAMzbtb10-3 | CDC27-D5: CCACAATATTCTAGATKCCAC | [^43^](http://sciwheel.com/work/citation?ids=8524955&pre=&suf=&sa=0) | 1148/200 |
|  | CDC27-R3: GCATTAGGAAATGAGCTCTTC | [^43^](http://sciwheel.com/work/citation?ids=8524955&pre=&suf=&sa=0) |  |
|  | CDC27-id: CCTTTCAGWTATAKWTCTTRTTG | This study |  |
|  | CDC27-ir: TATAAARAGCAWGTGGAKMTACAC | [^43^](http://sciwheel.com/work/citation?ids=8524955&pre=&suf=&sa=0) |  |
| Hodz3 | Hodz3-F: GGAGATCAGCAGYGGWGAGG | [^43^](http://sciwheel.com/work/citation?ids=8524955&pre=&suf=&sa=0) | 1060/80 |
|  | Hodz3-R: GCCACYTTRATGCAGTCCTC | [^43^](http://sciwheel.com/work/citation?ids=8524955&pre=&suf=&sa=0) |  |
|  | Hodz3-hF: CGGATATTAGCACCTGGAC | [^43^](http://sciwheel.com/work/citation?ids=8524955&pre=&suf=&sa=0) |  |
|  | Hodz3-hR: ACTCCTTTRCCAATCAGGG | [^43^](http://sciwheel.com/work/citation?ids=8524955&pre=&suf=&sa=0) |  |


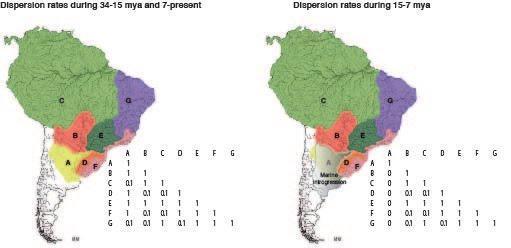


Fig. S1. Dispersal rates between the ecoregions over time used in the DEC analyses. The graphical representation of Miocene Marine introgression is approximated.


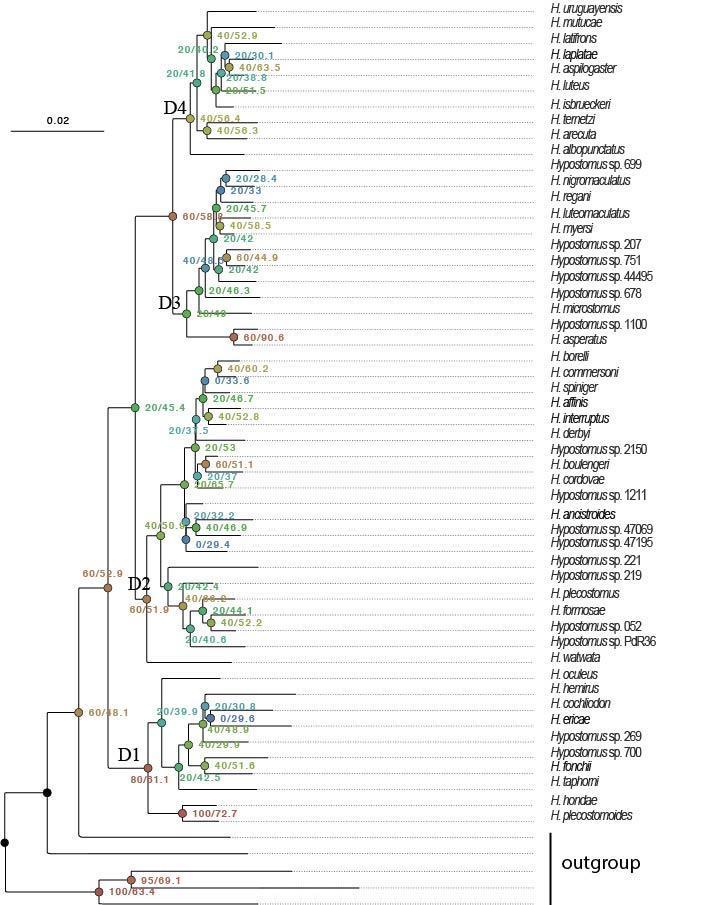


Fig S2. Phylogenetic reconstruction based on the concatenated data showing gCF / sCF in each branch estimated in IQtree. D1 – D4 are clade names (see main text).


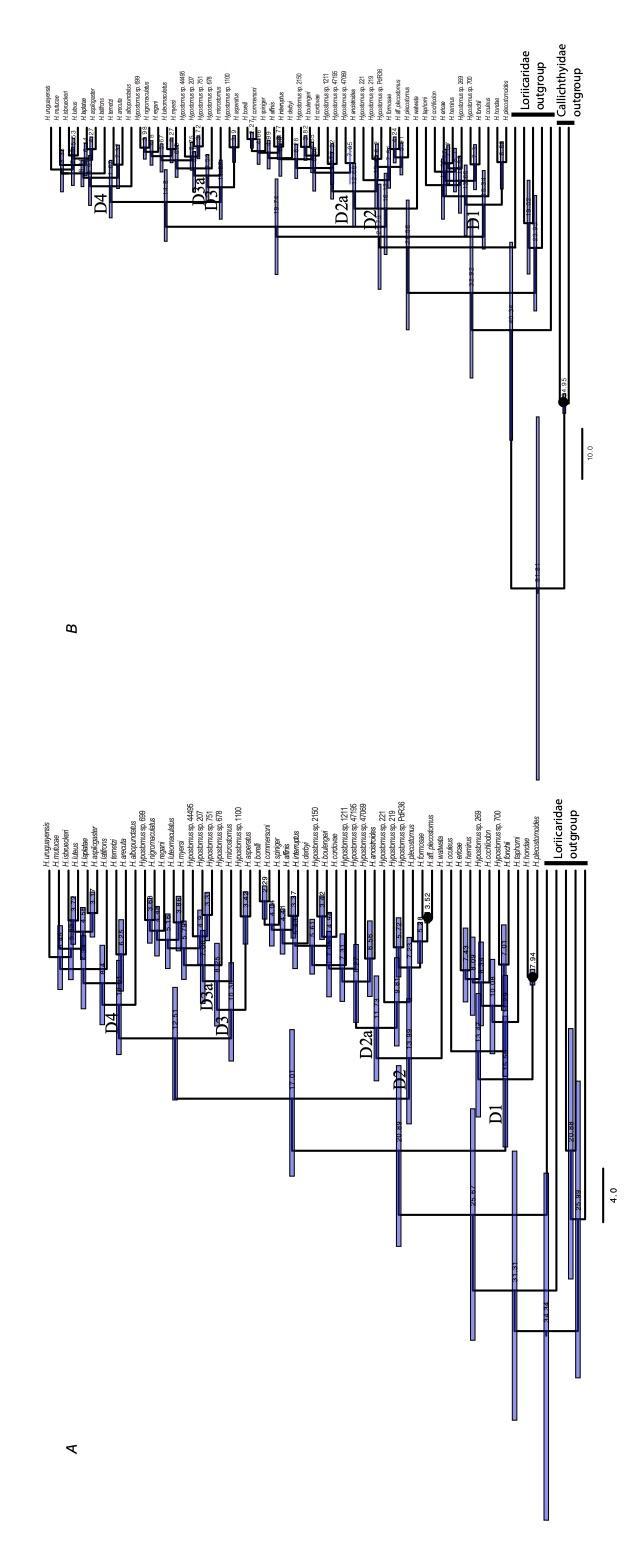


Fig. S3. Time-calibrated multilocus phylogeny for the *Hypostomus* genus, illustrating mean dates and confidence intervals (95% HPD) for each node. D1 – D4 are clade names (see main text). Three calibration analyses were performed using: (i) two dated hydrogeological events in A; (ii) a dated fossil in B; and (iii) the ages of the two hydrogeological events and the fossil (not shown). Black points are the calibrated nodes.

Table S3. Models of evolution for the habitat preference in *Hypostomus*. Nr: number of independent transition rates estimated; logLik: the maximum negative log-likelihood; AICc: Akaike information criterion corrected for sample size.

| Model | Nr | logLik | AICc | Δ-AICc |
| --- | --- | --- | --- | --- |
| ER: equal rates model | 1 | -64.199 | 130.470 | 1.996 |
| SYM: symmetric model | 10 | -51.846 | 128.475 | 0 |
| ARD: all rates different matrix | 20 | -48.074 | 159.481 | 31.006 |

List of the primary-source publications and web pages used in the inference of ancestral area analysis:

Publications:

Armbruster, J. W. 2004. Phylogenetic relationships of the suckermouth armoured catfishes (Loricariidae) with emphasis on the Hypostominae and the Ancistrinae. Zoological Journal of the Linnean Society v. 141 (no. 1): 1-80.

Cardoso, Y. P., Brancolini, F., Protogino, L. & Lizarralde, M. 2011. Actinopterygii, Siluriformes, Loricariidae, Hypostomus aspilogaster (Cope, 1894). Distribution extension and first record for Argentina. Check List 7, 596–598 (2011).

Cardoso, Y. P. et al. 2012. Origin of species diversity in the catfish genus Hypostomus (Siluriformes: Loricariidae) inhabiting the Paraná river basin, with the description of a new species. Zootaxa 83, 69–83.

Cardoso, Y. P. et al. 2016. Hypostomus formosae, a new catfish species from the Paraguay river basin with redescription of H. boulengeri (Siluriformes: Loricariidae). Ichthyological Exploration of Freshwaters 27, 9–23.

Cardoso, Y. P. et al. 2019. An integrated approach clarifies the cryptic diversity in Hypostomus Lacépède 1803 from the Lower La Plata Basin. An. Acad. Bras. Cienc. 91, e20180131 (2019).

Careaga, M., C. Ergueta, E. Gutiérrez, M. Maldonado and F. M. Carvajal-Vallejos 2020 (20 May) Inventario de los peces del río Yata (Beni, Bolivia). Hidrobiología Neotropical y Conservación Acuática v. 1 (no. 1): 69-80.

Knaack, J. 1999. A new species of suckermouth catfish (Hypostomus Lacépède 1803) from the Mato Grosso, Brazil (Pisces, Siluriformes, Loricariidae). Tropical Fish Hobbyist v. 47 (no. 11): 102-108.

Litz, T. & Koerber, S. 2014. Check List of the Freshwater Fishes of Uruguay ( CLOFF-UY ). Ichthyological Contributions of PecesCriollos 28, 1–40.

Ferraris, C. J., Jr. 2007. Checklist of catfishes, recent and fossil (Osteichthyes: Siluriformes), and catalogue of siluriform primary types. Zootaxa No. 1418: 1-628.

Mirande, J. M. and S. Koerber. 2015. Checklist of the freshwater fishes of Argentina (CLOFFAR). Ichthyological Contributions of PecesCriollos No. 36: 1-68.

Oliveira, R. R. de, F. R. V. Ribeiro, A. L. C. Canto and C. H. Zawadzki 2020. A new species of the Neotropical loricariid of Hypostomus cochliodon group (Hypostominae) from the lower Rio Tapajós basin, Brazilian Amazon. Journal of Fish Biology v. 97 (no. 2): 490-498 [1-9].

Oyakawa, O. T., A. Akama and A. M. Zanata. 2005. Review of the genus Hypostomus Lacépède, 1803 from rio Ribeira de Iguape basin, with description of a new species (Pisces, Siluriformes, Loricariidae). Zootaxa No. 921: 1-27.

Reis, R. E., S. O. Kullander and C. J. Ferraris, Jr. (eds). 2003. Check list of the freshwater fishes of South and Central America. CLOFFSCA. EDIPUCRS, Porto Alegre. 2003: i-xi + 1-729

Reis, R. E., C. Weber and L. R. Malabarba. 1990. Review of the genus Hypostomus Lacepéde, 1803 from southern Brazil, with descriptions of three new species (Pisces: Siluriformes: Loricariidae). Revue Suisse de Zoologie v. 97 (no. 3): 729-766.

Koerber, S., H. S. Vera-Alcaraz and R. E. Reis. 2017. Checklist of the fishes of Paraguay (CLOFPY). Ichthyological Contributions of PecesCriollos No. 53: 1-99.

Koerber, S. & Weber, C. 2014. The Hypostominae ( Siluriformes : Loricariidae ) of Argentina Ichthyological Contributions of PecesCriollos 29, 1–10.

Silva, G. S. C. et al. 2016. Transcontinental dispersal, ecological opportunity and origins of an adaptive radiation in the Neotropical catfish genus Hypostomus (Siluriformes: Loricariidae). Mol. Ecol. 25, 1511–1529.

Weber, C. and J. I. Montoya-Burgos. 2002. Hypostomus fonchii sp. n. (Siluriformes: Loricariidae)

from Peru, a key species suggesting the synonymy of Cochliodon with Hypostomus. Revue Suisse de Zoologie v. 109 (no. 2): 355-368.

Weber, C., R. Covain and S. Fisch-Muller. 2012. Identity of Hypostomus plecostomus (Linnaeus, 1758), with an overview of Hypostomus species from the Guianas (Teleostei: Siluriformes: Loricariidae). Cybium v. 36 (no. 1): 195-227.

Zawadzki, C. H. and P. H. Carvalho 2014. A new species of the Hypostomus cochliodon group (Siluriformes: Loricariidae) from the rio Aripuanã basin in Brazil. Neotropical Ichthyology v. 12 (no. 1): 43-51.

Zawadzki, C. H., H. P. da Silva and W. P. Troy. 2018 Redescription of Hypostomus latirostris (Regan, 1904) with the recognition of a new species of Hypostomus (Siluriformes: Loricariidae) from the upper rio Paraguay basin, Brazil. Ichthyological Exploration of Freshwaters v. 28 (no. 3) [no. IEF-1079]: [1-18] 253-270.

Zawadzki, C. H., L. F. C. Tencatt and H. A. Britski. 2019. Taxonomic revision of Hypostomus albopunctatus (Siluriformes: Loricariidae) reveals a new piece of the Hypostomus jigsaw in the upper Río Paraná basin. Journal of Fish Biology v. 96 (no. 1): 1-13 [230-242].

Web pages:

<http://researcharchive.calacademy.org/research/ichthyology/catalog/fishcatmain.asp>).

<http://www.pecesargentina.com.ar/base_peces/login.php>

<http://silurus.acnatsci.org/>

<https://www.planetcatfish.com/>

Fig. S4 . Calibrated tree shows the posterior probabilities for the ecoregion assignments at each ancestral node performed in RevBayes. Number above branches are posterior probabilities of the start states (squares in Fig. 1) and number next to the nodes are posterior probabilities of the end state (circles in Fig. 1).


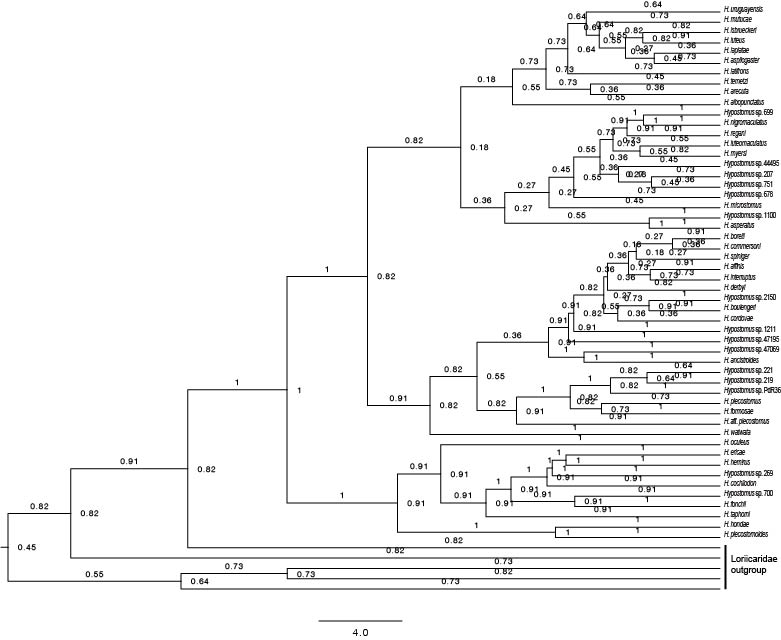


Fig. S5. The number of diversification events for each clade in time windows of one Mya, as well as for the total number of speciation events in *Hypostomus* within La Plata Basin. We also plotted the
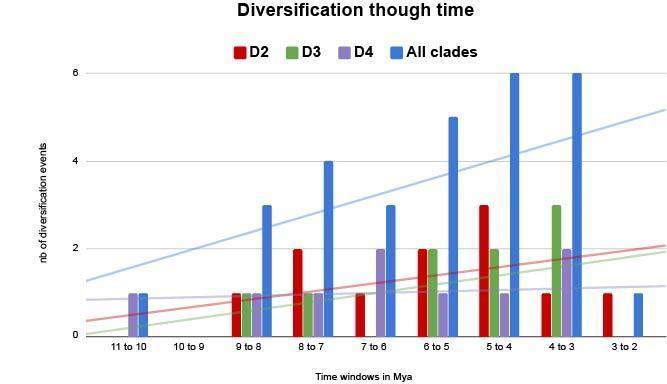
trend line of each data.
